# Great tits differ in glucocorticoid plasticity in response to spring temperature

**DOI:** 10.1098/rspb.2022.1235

**Published:** 2022-11-09

**Authors:** Michaela Hau, Caroline Deimel, Maria Moiron

**Affiliations:** ^1^ Max Planck Institute for Ornithology, Seewiesen, Germany; ^2^ University of Konstanz, Konstanz, Germany; ^3^ Institute of Avian Research, Wilhelmshaven, Germany

**Keywords:** glucocorticoid hormones, reaction norm approach, environmental temperature, climate change, individual differences, hormonal plasticity

## Abstract

Fluctuations in environmental temperature affect energy metabolism and stimulate the expression of reversible phenotypic plasticity in vertebrate behavioural and physiological traits. Changes in circulating concentrations of glucocorticoid hormones often underpin environmentally induced phenotypic plasticity. Ongoing climate change is predicted to increase fluctuations in environmental temperature globally, making it imperative to determine the standing phenotypic variation in glucocorticoid responses of free-living populations to evaluate their potential for coping via plastic or evolutionary changes. Using a reaction norm approach, we repeatedly sampled wild great tit (*Parus major*) individuals for circulating glucocorticoid concentrations during reproduction across five years to quantify individual variation in glucocorticoid plasticity along an environmental temperature gradient. As expected, baseline and stress-induced glucocorticoid concentrations increased with lower environmental temperatures at the population and within-individual level. Moreover, we provide unique evidence that individuals differ significantly in their plastic responses to the temperature gradient for both glucocorticoid traits, with some displaying greater plasticity than others. Average concentrations and degree of plasticity covaried for baseline glucocorticoids, indicating that these two reaction norm components are linked. Hence, individual variation in glucocorticoid plasticity in response to a key environmental factor exists in a wild vertebrate population, representing a crucial step to assess their potential to endure temperature fluctuations.

## 1. Introduction

Fluctuations in key climatic variables like environmental temperature can affect the energy balance of vertebrate populations [1,2]. Individuals from wild populations respond by displaying phenotypic plasticity (or ‘flexibility’), to reversibly and repeatedly adjust their expression of thermoregulatory, metabolic, and behavioural traits [3,4]. Glucocorticoids, a conserved set of vertebrate steroid hormones, transmit information about environmental variation like temperature fluctuations by rapidly changing their concentrations in the blood [5–7]. Glucocorticoid receptors are distributed in various parts of the brain and body [8–10], enabling these hormones to function as higher-order regulators to pleiotropically promote phenotypic plasticity [11–13]. For these reasons, the plasticity of glucocorticoid responses has been suggested as one of the mechanisms underlying within- and among-individual differences in phenotypic plasticity [13–16].

Individuals can differ in how strongly they respond to variation in environmental temperature in plastic changes of metabolic rate [17,18] or foraging activity [19]. Quantifying such individual differences in the glucocorticoid response in wild vertebrate populations is crucial for evaluating the mechanisms shaping within- and among-individual differences in phenotypic plasticity and to elucidate their evolutionary potential (e.g. [13,14–16,20,21]). Alterations in environmental temperatures across the globe are one of the most prominent manifestations of the ongoing climate change [22]. The predicted increases in yearly average temperatures and in the occurrence of temperature extremes will probably alter the selection pressures that wild populations are exposed to. It is expected that populations which exhibit among-individual variation in plastic responses to environmental temperature are better equipped to withstand climate change and if the standing phenotypic variation has a heritable component, may evolutionarily adapt to these changes (e.g. [23,24]).

To our knowledge, it is presently unknown whether individuals from free-living vertebrate populations differ in their glucocorticoid plasticity when experiencing natural fluctuations in environmental temperature. Addressing this question requires a reaction norm approach, which in its simplest form describes a linear relationship between an individual's repeatedly measured phenotype (e.g. glucocorticoid concentrations) and an environmental or internal gradient (e.g. environmental temperature) [25,26]. Using linear mixed models, phenotypic plasticity can be decomposed into the *elevation* (the average value of a trait in the average environment), the *slope* of the response to the environment (the change in this trait across the gradient, i.e. its plasticity), and their covariance (i.e. whether links exist between these two reaction norm components that may constrain their variation) [25,26]. These hierarchical analyses allow to separately quantify glucocorticoid elevation (average concentrations) and slope (plastic changes) for each individual (within-individual level), while also estimating whether differences among individuals from this population exist in average concentrations and/or their plasticity to a gradient in environmental temperature.

Reaction norm analyses can be used to analyse glucocorticoid variation separately for baseline and stress-induced concentrations, and to test whether they covary. While glucocorticoid concentrations in individuals going about their lives hardly ever are ‘basal’ and at the lowest possible level, samples taken within 2–3 min of capture, the time lag of the hypothalamo-pituitary-adrenal (HPA) axis to respond to a disturbance, are usually referred to as ‘baseline’ and considered to reflect normal daily activities [27–29]. By contrast, ‘stress-induced’ concentrations indicate samples taken 30–60 min after a major disturbance that activated the HPA axis and resulted in a major increase in blood glucocorticoid concentrations [30]. Separate analyses at these distinct concentrations are justified because glucocorticoids activate different genomic receptors and can exert divergent phenotypic effects [30–33]. Comparative work across vertebrate taxa also suggests that baseline and stress-induced corticosterone concentrations are shaped by different selection pressures [34]. At low baseline levels, circulating glucocorticoid concentrations exert their actions by binding to the high-affinity mineralocorticoid receptor to promote glucose availability to tissues, stimulating appetite, and regulating protein and lipid stores [30,31]. This supports predictable variations in energy demands in response to changes in weather, life-history stage, and time of day, thereby promoting plastic adjustments in behavioural and physiological traits like foraging, offspring provisioning, or metabolic rate [31]. Whenever conditions change unpredictably, inducing major energetic or psychological challenges, glucocorticoids increase within a few minutes to reach high stress-induced concentrations, which then also occupy the low-affinity glucocorticoid receptor [30,32,35]. This mechanism shifts an individual into an ‘emergency state’, which can involve mobilization of its energy stores, re-allocation of energetic resources to processes that facilitate coping with and recovering from the immediate challenge, and the inhibition of reproductive behaviours that are not essential for surviving the challenge [12,30].

Recent work suggests that individual differences in glucocorticoid reaction norm elevation and slope exist in wild vertebrate populations (summaries: [14,16,21]). However, some of these studies determined glucocorticoid metabolites from excrements (urine: [36], faeces: [37]), which poses several biological and methodological challenges that make the interpretation of results and comparisons across species and studies difficult (reviews: [38,39]). So far, studies in the wild that measured blood glucocorticoid concentrations focused on aspects of the ‘stress-response’, i.e. the increase from baseline to stress-induced concentrations [40,41]. Some studies in captivity documented individual variation in circulating baseline glucocorticoid plasticity (in birds; [42,43]), while others found individual differences only in glucocorticoid elevation (in water-borne hormone samples of fishes; [44,45]). Findings in captivity can be of limited relevance for natural situations because individuals may be chronically stressed and/or lack natural cues, which can bias glucocorticoid concentrations (e.g. [46]).

We therefore conducted a 5-year study in a population of free-living great tits (*Parus major*) in which we applied a reaction norm approach to determine within- and among-individual variation in corticosterone plasticity (the main avian glucocorticoid) along a natural gradient in environmental temperature. We repeatedly sampled adults of both sexes for circulating concentrations of baseline and stress-induced corticosterone using a standardized capture-restraint protocol [27] during a specific reproductive stage, the peak nestling provisioning phase. Repeated sampling mostly occurred across years, since in our population great tits infrequently initiate a second clutch. Using a series of general linear-mixed effect models (GLMMs), we tested the following predictions:

(i) baseline and stress-induced corticosterone concentrations increase with lower environmental temperature at the population level, as is commonly found in endotherms (summaries: [5,6,7,23]). Baseline and stress-induced corticosterone concentrations are predicted to be better associated with short-term (temperature at capture) than with longer-term temperature measurements (temperature averages on capture day or on days preceding capture) because small endotherms should track short-term temperature changes as they usually carry little energy reserves;
(ii) average (elevation of the reaction norm) baseline and stress-induced corticosterone concentrations differ among individuals (e.g. meta-analyses: [47,48]);
(iii) within individuals, corticosterone concentrations change plastically and increase with lower environmental temperatures, especially when these are below the thermoneutral zone (the range of environmental temperatures at which individuals do not expend energy on thermoregulation; approximately 15–30°C in great tits [49,50]);
(iv) the reaction norm slope (plasticity) in response to environmental temperature differs among individuals, with some individuals showing greater corticosterone plasticity (steeper slope) than others (e.g. [42]);
(v) elevation and slope covary among individuals as in previous work [44]. This has rarely been tested (summarized by: [16]); and
(vi) baseline and stress-induced corticosterone concentrations show positive covariation at the population and within-individual levels, but not at the among-individual level (e.g. [51]). Population-level correlations, i.e. higher baseline concentrations at capture being associated with higher stress-induced concentrations, could arise from within-individual processes like a concerted change in both traits because an individual's overall state is altered by environmental temperature. A non-exclusive alternative are among-individual processes, i.e. when an individual consistently displays higher concentrations in both corticosterone traits compared to conspecifics, owing to genetic, maternal, or environmental factors (e.g. [51]).

## 2. Methods

### (a) Field work

Individually marked adult great tits were studied in a free-living nestbox population in the Ettenhofer Holz, Upper Bavaria, Germany from early May to early July of 2015 through to 2019. The birds were captured when entering the nestbox to feed their 8-day-old nestlings (hatching = day 0; further details in the electronic supplementary material).

#### (i) Temperature recordings

Temperature at capture was recorded using a handheld thermometer (BASETech Thermometer E0217, accuracy ±1°C). Whenever temperature could not be directly recorded at the capture location, we used data from a HOBO weather station (details in the electronic supplementary material) located within our study area. In that case, we used the temperature of the 30 min increment recorded by this station closest to the time of capture. Mean, maximum, or minimum temperatures used in the statistical analyses were calculated from the weather station data.

#### (ii) Blood sampling

Blood was sampled for baseline corticosterone within about 3 min of blocking of the entrance hole of the nest by pricking the wing vein with a 26 g needle and collecting the blood in heparinized microcapillaries. The time to finish blood sampling for baseline corticosterone was (mean ± s.d.) 2.1 ± 0.58 min, range: 0.92 to 4 min. After the blood sampling, birds were kept in an opaque cotton bag until banding, biometric measurements (mass, tarsus and wing length, muscle and fat scores) and a second blood sample for stress-induced corticosterone was taken about 30 min after initial capture (mean ± s.d.: 30.1 ± 2.88 min, range: 19.75 to 45 min). Following the second blood sample, we returned the bird into the nestbox. For further details on blood sampling and hormone analysis using enzyme-immunoassays see the electronic supplementary material.

### (b) Statistical analyses

The dataset for baseline corticosterone contained 389 measurements from 240 individuals. Among them, 140 individuals had one measurement, 68 individuals had two, 19 had three, nine had four and four individuals had five. For stress-induced corticosterone, the dataset contained 376 measurements from 237 individuals. Among them, 144 individuals had one measurement, 62 individuals had two, 19 had three, nine had four and three individuals had five. Most repeated measures were obtained in different years (further details in the electronic supplementary material). We included data from all individuals in all our analyses, i.e. also those that were sampled only once. This methodology improves power while yielding similar variance estimates with narrower confidence intervals [52]. For all models, we visually inspected the residuals of the data and found them to qualitatively follow standard criteria for residual normality.

#### (i) Population-level corticosterone association with temperature measures (model 0)

We ran a series of univariate GLMMs to identify the temperature measure (temperature at the moment of capture, and mean, minimum and maximum temperatures on the day of capture, on the day prior to capture, and on the three days prior to capture) that explained baseline and stress-induced corticosterone, respectively, best in this population of great tits. All models included the fixed and random effects described for model 0 in table 1. We then used deviance information criterion (DIC) model comparison to identify the best model (the one with the lowest DIC value) and therefore the best temperature proxy for each corticosterone level (but for DIC criticism see : [53,54].

**Table 1.** Overview and structure of different statistical models (numbered sequentially) used to address the main research questions in this study. (ID, identity.)

	research question	fixed effects	random effects	residuals
model 0	population-level corticosterone association with temperature measures^a^	sex, year of sampling, bleeding time, temperature metric at different scales	ID: random intercepts	homogeneous
model 1	population-level corticosterone responses to temperature and individual differences in corticosterone concentrations ^a^	sex, year of sampling, bleeding time, temperature at capture	ID: random intercepts	homogeneous
model 2	within-individual corticosterone changes in response to temperature^a^	sex, year of sampling, bleeding time, average temperature at capture, temperature observation's deviation	ID: random intercepts	homogeneous
model 3	individual differences in corticosterone plasticity^a^	sex, year of sampling, bleeding time, temperature at capture	ID: random intercepts & slopes	heterogeneous (5)
model 4	correlations between baseline and stress-induced corticosterone^a^	sex, year of sampling, bleeding time, temperature at capture	ID: random intercepts	homogeneous

^a^Either baseline or stress-induced corticosterone were fitted as response variable, respectively, and modelled assuming a Gaussian error and dividing concentrations by 1000 to facilitate model convergence.

#### (ii) Population-level responses to temperature and individual differences in corticosterone concentrations (model 1)

To understand the factors that explain the population-level variation in baseline and stress-induced corticosterone, we built two univariate GLMMs for baseline and stress-induced corticosterone, respectively, assuming a Gaussian error distribution and dividing concentrations by 1000 (to facilitate model convergence; as in all models). As fixed effects, we fitted sex (factor with two levels: male or female), year of sampling (factor with five levels: 2015–2019, to control for among-year differences), bleeding time (continuous variable, to control for the time needed to extract a blood sample, see data range above) and temperature at capture (continuous variable ranging from 3.8 to 26.8°C) (model 1, table 1). Bleeding time and temperature at capture were mean-centred and variance standardized to facilitate the interpretation of their relative influence on baseline and stress-induced corticosterone. In each model, we also fitted random intercepts for individual identity to control for repeated measures of individuals across years and estimate the variance explained by among-individual differences and the residual variance. Repeatability of each trait, conditional to the variance explained by the fixed effects, was estimated as the proportion of the total phenotypic variance explained by individual variance.

#### (iii) Within-individual corticosterone changes in response to temperature (model 2)

To analyse whether individuals changed corticosterone concentrations over their sampling period in response to environmental temperature, we used a ‘within-subject centring’ approach (i.e. [55,56]). We calculated: (i) the average value of temperature that each individual has experienced, and (ii) the observation's deviation from the focal individual's average value. As such, ‘average temperature’ represents the among-individual temperature effect, while ‘observation's deviation’ represents the within-individual plastic change of corticosterone with temperature [56]. We then built two univariate GLMMs where either baseline or stress-induced corticosterone were fitted as response variable, and the among- and within-individual components of temperature were fitted as fixed effects. We modelled the same fixed and random effect structure as in model 1 (see model 2 in table 1). Finally, we tested whether the among- and within-individual effects of temperature at capture on corticosterone differed statistically. A difference between the within- and between-individual effects that is close to zero and with 95% credible intervals (CI) largely overlapping zero suggests that the within-individual, plastic response to temperature would be sufficient to explain the overall population-level response to variation in temperature. To do so, we calculated the difference between the parameter estimates of the within- and among-individual effect of temperature, and assessed whether their 95% CI overlapped zero.

#### (iv) Individual differences in corticosterone plasticity (model 3)

To test whether baseline and stress-induced corticosterone showed differences among individuals in their plastic response to environmental temperature at capture (individual by environment interactions (I x E)), we used a random regression analysis [25]. We fitted two univariate GLMMs with baseline and stress-induced corticosterone as response variables, respectively. We modelled the same structure of fixed and random effects as in model 1 while adding the interaction of individual identity with temperature as a random slope effect (see model 3 in table 1). With this model, we tested for among-individual variance in the elevation and slopes of corticosterone concentrations in response to temperature at capture, while also testing for the covariance and correlation among individuals' elevation and slopes. Inappropriate modelling of residual variance (e.g. assuming residual homogeneity) can lead to erroneous inferences of slope variance in random regression models (for further details see [57]. Hence, we assumed residual effects to be year-specific (i.e. estimated residual variance for each of the five study years) and uncorrelated across years (i.e. diagonal residual error structure). To assess whether models with (i) both random intercept and random slope terms, and/or (ii) with heterogeneous residual structure explained the data better than models with only random intercepts and/or homogeneous residuals, we used DIC model comparison (being the best-fitting model the one with the lowest DIC value, but see [53,54] for criticism on DIC model comparisons).

#### (v) Correlations between baseline and stress-induced corticosterone (model 4)

We fitted a bivariate GLMM to investigate whether and how baseline and stress-induced corticosterone covaried at the population (phenotypic), among- and within-individual levels. To do so, we simultaneously modelled baseline and stress-induced corticosterone as response variables. We fitted the same fixed and random effect structure as described in model 1, while also fitting unstructured covariance matrices for the random effect individual identity and residual variance (see model 4 in table 1). We obtained correlations by dividing the covariance of two traits with the product of their variances.

#### (vi) Statistical model implementation

All statistical models were fitted using a Bayesian framework implemented in the statistical software R (v. 3.6.1, R Core Team 2019) using the R-package MCMCglmm [58]. For all models, we used parameter-expanded priors [58]. The number of iterations and thinning interval were chosen for each model to ensure that the minimum Markov chain Monte Carlo effective sample sizes for all parameters were 1000. Burn-in was set to a minimum of 5000 iterations. The retained effective sample sizes yielded absolute autocorrelation values lower than 0.1 and satisfied convergence criteria based on the Heidelberger and Welch convergence diagnostic. We drew inferences from the posterior modes and 95% CI. We considered fixed effects and correlations to be important if the 95% CI did not include zero; estimates centred on zero were considered to provide support for the absence of an effect.

## 3. Results

### (a) Population-level responses to temperature, and individual differences in average concentrations

**Figure 1.**
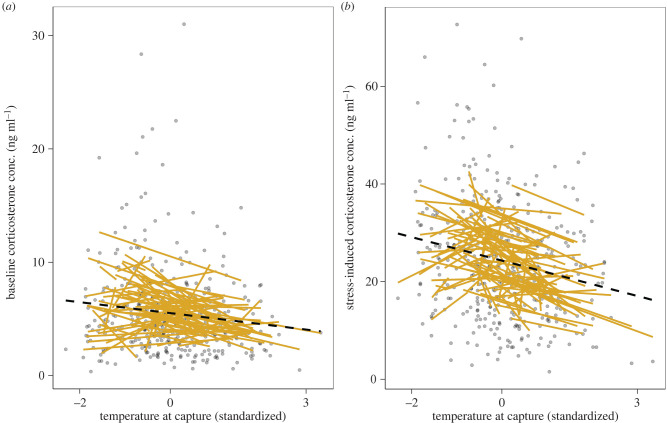
Predictions from two random regression models of baseline (*a*) and stress-induced (*b*) corticosterone concentrations in response to environmental temperature at capture (mean-centred and variance standardized). Each yellow solid line represents a single individual, the dashed black line represents the population-level response to temperature, and grey dots represent the raw phenotypic data (colours are provided in the online version of the figure).

Variation in baseline and stress-induced corticosterone was best explained by environmental temperature at the moment of capture (DIC model comparisons of model 0, second-best model with maximum temperature at day of capture: ΔAIC > 4 for baseline and stress-induced corticosterone, respectively; electronic supplementary material, tables S1 and S2). Baseline and stress-induced corticosterone were both negatively related to temperature at capture (model 1, table 2, figure 1), i.e. baseline and stress-induced corticosterone levels were higher when temperatures at capture were lower (range of variation: approx. 4–27°C, electronic supplementary material, figures S1 and S2). This significant effect of temperature at capture was somewhat stronger for stress-induced corticosterone than for baseline corticosterone (table 2, figure 1), indicating that stress-induced corticosterone might be even more conditioned by local temperatures than baseline concentrations. Furthermore, as expected from ample previous work including on great tits [27–29,59], baseline, but not stress-induced corticosterone, was influenced by the time needed to complete a blood sample, with longer sampling times being associated with higher concentrations of baseline corticosterone. We also found large year differences, and, to a lower extent, sex differences for both hormonal traits (model 1, table 2). Both hormonal traits were repeatable: individual differences in average trait expression explained 17% of the total population variance in baseline corticosterone, and 26% in stress-induced corticosterone (table 2).

**Table 2.** Sources of phenotypic variation in baseline and stress-induced corticosterone concentrations (model 1). (Both variables were divided by 1000 and modelled following a Gaussian error distribution. Estimates of fixed (*β*) and random (*σ*^2^) parameters are shown as posterior modes with 95% credible intervals (CI). Repeatability, conditional to the variance explained by the fixed effects, was estimated as the proportion of the total phenotypic variance explained by individual variance.)

fixed effects	baseline corticosterone		stress-induced corticosterone	
	*β*	95% CI	*β*	95% CI
intercept	6.817	[5.935, 7.696]	23.904	[21.071, 26.323]
sex [female]	0.696	[−0.064, 1.489]	2.308	[−0.038, 4.726]
year [2016]	−1.602	[−2.663, −0.622]	−6.404	[−9.673, −3]
year [2017]	−1.891	[−2.91, −0.826]	−1.634	[−4.887, 1.571]
year [2018]	−1.252	[−2.329, −0.095]	7.52	[3.882, 11.196]
year [2019]	−3.452	[−4.641, −2.243]	−0.604	[−4.06, 2.941]
bleeding time (scaled)	1.022	[0.609, 1.404]	0.561	[−0.644, 1.799]
temperature at capture (scaled)	−0.678	[−1.006, −0.275]	−2.78	[−3.939, −1.716]
**random effects**	***σ*2**	**95% CI**	***σ*2**	**95% CI**
individual	2.141	[0.05, 4.119]	32.8	[13.372, 53.256]
residual	10.42	[8.047, 12.824]	93.496	[74.123, 115.069]
repeatability	0.169	[0.01, 0.313]	0.258	[0.122, 0.405]

### (b) Within-individual corticosterone changes in response to temperature

Both baseline and stress-induced corticosterone showed significant within-individual variation in response to environmental temperature (*β*_within−ID temperature effect_ = −0.984 [−1.481, −0.48] for baseline levels, and *β*_within-ID temperature effect_ = −2.319 [−4.023, −0.818] for stress-induced levels; model 2; electronic supplementary material, table S3). This indicates that individual great tits plastically adjusted their hormonal levels from one sampling event to another in response to variation in temperature (within-individual plasticity). There was also between-subject variation (*β*_between-ID temperature effect_) in baseline and stress-induced corticosterone (although the effect for baseline corticosterone is slightly overlapping zero, model 2; electronic supplementary material, table S3). However, there was no significant difference between the within- and between-subject effects for either baseline (posterior mode *β*_B_–*β*_W_ = 0.567, 95% CI = −0.114, 1.289) or stress-induced corticosterone (posterior mode *β*_B_–*β*_W_ = −0.673, 95% CI = −3.073, 1.231), suggesting that a single mechanism explains the observed patterns: plastic responses of individuals to temperature variation underlie the population-level relationship with temperature for both hormonal traits.

### (c) Among-individual differences in corticosterone plasticity and elevation-slope covariance

For both baseline and stress-induced corticosterone, DIC model comparison showed that the best-fitting model included a random factor for the interaction between individual identity and temperature at capture. This model also allowed residual variation to differ among the five years of sampling (i.e. heterogeneous residual variance; model 3; electronic supplementary material, table S4). These results imply that individuals differ in average baseline and stress-induced corticosterone at the population's average temperature (i.e. among-individual variation in elevation) as well as in baseline and stress-induced corticosterone plasticity, with some individuals responding more strongly to temperature changes than others (i.e. among-individual variation in slopes; table 3, figure 1; electronic supplementary material, figure S2). For baseline corticosterone, we found a negative covariation of elevation and slope, indicating a ‘fanning-in’ pattern in phenotypic variation along the temperature gradient (figure 1, table 3).

**Table 3.** Results from a random regression model of baseline and stress-induced corticosterone as a function of environmental temperature at capture (model 3). (Estimates of fixed (*β*) and random (*σ*^2^) parameters are shown as posterior modes with 95% credible intervals (CI).)

fixed effects	baseline corticosterone		stress-induced corticosterone	
	*β*	95% CI	*β*	95% CI
intercept	6.83	[5.593, 8.16]	24.03	[21.224, 26.999]
sex [female]	0.69	[0.025, 1.226]	2.43	[0.194, 4.912]
year [2016]	−1.67	[−3.055, −0.364]	−6.43	[−10.053, −2.803]
year [2017]	−1.91	[−3.263, −0.548]	−1.33	[−4.588, 2.077]
year [2018]	−1.24	[−2.632, 0.302]	7.35	[3.26, 11.122]
year [2019]	−3.38	[−4.78, −2.097]	−0.74	[−3.805, 2.544]
bleeding time (scaled)	1.00	[0.672, 1.296]	0.75	[−0.411, 1.902]
temperature at capture (scaled)	−0.62	[−0.856, −0.353]	−2.39	[−3.545, −1.284]
**random effects**	***σ*2**	**95%CI**	***σ*2**	**95%CI**
individual				
intercept	1.90	[0.804, 3.061]	41.87	[22.058, 60.442]
linear slope	0.29	[0.001, 0.658]	3.60	[0.009, 10.882]
intercept–slope covariance	−0.47	[−0.957, 0.012]	−2.88	[−9.884, 2.937]
intercept–slope correlation	−0.65	[−0.987, −0.207]	−0.28	[−0.895, 0.352]
residual 1	30.88	[20.679, 41.038]	111.31	[66.323, 159.672]
residual 2	6.21	[3.768, 8.923]	102.74	[65.293, 141.702]
residual 3	5.96	[3.96, 8.473]	81.00	[45.969, 112.406]
residual 4	6.42	[3.671, 9.286]	102.66	[58.087, 153.517]
residual 5	1.15	[0.277, 2.318]	15.26	[0.281, 38.185]

### (d) Covariation between baseline and stress-induced corticosterone concentrations

We found a significant positive correlation between baseline and stress-induced corticosterone concentrations at the population level (*r* = 0.326, 95% CI [0.223, 0.407], model 4; electronic supplementary material, table S5), indicating that higher concentrations of baseline corticosterone were associated with higher concentrations of stress-induced corticosterone. We also observed a positive correlation at among- and within-individual levels (among: *r* = 0.35 [0.144, 0.738], within: 0.25 [0.102, 0.388]; electronic supplementary material, table S5). The positive correlation among individuals indicates that birds with on average higher baseline corticosterone concentrations also had on average higher stress-induced corticosterone than others. The positive within-individual correlation means that when a bird exhibited high baseline corticosterone concentrations at a given sampling event, it also exhibited high stress-induced corticosterone concentrations.

## 4. Discussion

We applied a reaction norm approach to quantify variation in circulating glucocorticoid concentrations in free-living great tits sampled repeatedly across different breeding events over five years. As predicted, individuals from our study population plastically changed circulating baseline and stress-induced corticosterone concentrations in response to the environmental temperature they experienced at capture in each year during the peak nestling provisioning phase (figure 1; electronic supplementary material, figure S2). Importantly, our analyses revealed that individuals differed in both reaction norm components for baseline and stress-induced corticosterone concentrations: in average concentrations (i.e. reaction norm elevations) and in their plasticity (reaction norm slopes), with some individuals changing corticosterone concentrations more strongly in response to environmental temperature than others (figure 1; electronic supplementary material, figure S2). These findings provide unique evidence that plastic endocrine responses to a key ecological factor vary among and within individual free-living great tits. Thus, if individual variation in glucocorticoid plasticity was heritable (e.g. [60,61]) and under selection, it would provide the basis for evolution to occur in a conserved higher-order mechanism that enables vertebrates to adjust their phenotype to fluctuations in climatic conditions [7,21,23,62].

Our prediction that corticosterone concentrations are higher at lower environmental temperatures at the population level was confirmed, in line with some earlier findings (summaries: [5–7]). Importantly, we found that this negative relationship was explained by within-individual plasticity. A likely mechanism is increased energetic demands associated with thermoregulation that can lead to within-individual increases in corticosterone at lower temperatures (summaries: [23,62]). In our study, temperatures at capture varied between approximately 4–27°C (electronic supplementary material, figures S1 and S2). Assuming that the lower limit of the thermoneutral zone in our great tit population is around 15°C [49,50], we collected two-thirds of the baseline and stress-induced corticosterone samples at temperatures that required the birds to produce heat to maintain body temperature. Two pathways underlying this response are conceivable. First, cooler environmental temperatures may have induced increases in corticosterone concentrations directly via thermoreceptors relaying information to hypothalamic brain areas (reviewed in [23,63]). Second, an indirect pathway could involve metabolic rate, which probably was higher in individuals that needed to generate heat through shivering and non-shivering thermogenesis [63]. Indeed, experimentally induced increases in metabolic rate are positively associated with corticosterone concentrations within and among individual zebra finches (*Taeniopygia guttata*) [64,65]. Metabolic rates may also have been increased at lower temperatures because great tits had to exhibit greater foraging activity to provision their young as insect food is harder to find and less abundant (e.g. [66]).

At present, we cannot exclude that other factors associated with environmental temperature, for example, precipitation, wind, or barometric pressure contributed to within-individual corticosterone plasticity [5,6]*.* Furthermore, because most of the repeated sampling occurred across years, variables unrelated to environmental temperature could also have contributed to within-individual plasticity in corticosterone. These include an individual's experiences within a year that may have altered its perception, processing and responses to environmental challenges, for example via epigenetic modifications of both HPA axis (and thus corticosterone release) and receptor functioning (e.g. [67,68]). To address these questions, future work should sample individuals at shorter time scales like across seasons, life-history stages, or even days that vary in environmental temperatures. Moreover, experimental manipulations of environmental temperatures and/or an individual's thermoregulatory abilities will be important to ascertain causal relationships.

As expected, individuals in our study also differed from each other in average baseline and stress-induced concentrations (i.e. reaction norm elevation, table 2 and figure 1). Our corticosterone repeatability estimates (baseline: 17%; stress-induced: 26%) are within the range of those reported in recent meta-analyses (across vertebrate taxa, baseline: 18–23%; stress-induced: 37–38%; [47,48]). At the same time, our estimates suggest that a substantial percentage of the variance remains unexplained (residual variance), which could in principle result from high within-individual variation, low among-individual variation, or high measurement error acting singly or in combination [47]. Given our long sampling intervals, we consider changes in environmental or internal conditions of an individual a likely source of high residual variance, as shorter sampling intervals tend to increase repeatability estimates for glucocorticoid traits [47,48]. Nevertheless, our repeatability estimates align well with heritability estimates in studies on free-living bird populations, where additive genetic effects explained less than 20% of the variance in baseline and more than 30% in stress-induced concentrations, respectively [60,69,70].

The presence of I × E in our great tit population, i.e. among-individual differences corticosterone plasticity at both levels (figure 1, table 3), is intriguing from a proximate viewpoint. In principle, individual differences in plasticity (reaction norm slope) could arise from two non-exclusive processes: (i) long-term individual differences that are based on genetic information, maternal or other early developmental effects; or (ii) short-term variation in an individual's state—or both (e.g. [71,72]). Although we sampled great tits during a standardized parental phase, individuals could differ in various aspects, including body condition, health status, territory or partner quality, predation pressure, reproductive investment and age (sexes differed in some aspects, see table 3; electronic supplementary material, table S3, but sample size limitations did not allow us to test whether sexes differed in plasticity). Both long- and short-term processes could generate among-individual differences in glucocorticoid slopes via variation in, for example, the perception and neuro-endocrine processing of environmental temperature, the capacity to synthesize glucocorticoids (including the sensitivity of the HPA axis to releasing hormones), the binding of glucocorticoids to carrier molecules in the blood, their enzymatic deactivation, and differences in receptor densities [13,14,73]. Many short- or long-term variables can also determine an individual's energy intake and/or usage and thus affecting circulating glucocorticoid concentrations [35,74]. These hypotheses can be tested experimentally.

Elevation and slope of reaction norms in baseline corticosterone were correlated within individuals, suggesting that these two features of the endocrine response to environmental temperature are linked in our free-living great tit population (table 2). The fanning-in pattern evident in baseline corticosterone slopes (figure 1*a*) suggests a reduction (canalization) in phenotypic variance at higher temperatures. At present, we can only speculate that differences in the individuals' energetic or health state may play a role. Glucocorticoids have context-, state- and tissue-dependent effects: they can mobilize energy reserves in adipose tissue but also increase fat storage in the liver (reviewed in [31]). Thus, future work needs to assess an individual's energetic state (e.g. via morphometric, or blood and tissue composition measures), ideally coupled with a manipulation of energetic challenges. From an evolutionary viewpoint, the covariation between elevation and slope is important because it suggests that these two attributes of baseline corticosterone are somehow linked [21].

Finally, great tit individuals showed a negative relationship in both baseline and stress-induced corticosterone with environmental temperature (table 3), suggesting a concerted response in these two traits to the same environmental factors. The positive covariation between baseline and stress-induced corticosterone at the population, among-individual and within-individual level (electronic supplementary material, table S5) supports this view. A likely scenario is that environmental temperature affects an individual's allostatic load or reactive homeostasis (i.e. the cumulative challenges, energetic or otherwise, that an individual has to cope with at one point in time; [35,74]), and this change in state could influence both traits—also in different populations and species (e.g. [34,75]).

## 5. Conclusion

This line of work is important for evaluating the consequences of climate change for wild vertebrate populations. Our reaction norm study suggests that individuals from a wild bird population plastically change glucocorticoid concentrations in response to a key environmental gradient – temperature – and that they differ in the extent of their glucocorticoid plasticity. These results are vital for a refined understanding of the physiological processes that underlie animal–environment interactions as well as the evolutionary forces that may shape them. As next steps, we should test the generality of these findings for a wider range of species and environmental factors and connect individual variation in glucocorticoid reaction norm components to expressed phenotypes like reproductive success, recruitment and survival, and study patterns of selection [13–15,21]. Moreover, the integration of plastic glucocorticoid responses with multiple other traits will be a rewarding future avenue, as it has the potential to provide important insights into the evolutionary patterns underlying multivariate plasticity (i.e. ‘integration of plasticity’, [21,76]), and overall, the evolution of reversible phenotypes. Although reaction norm studies require large sample sizes both in number of individuals measured and observations per individual, other areas in biology can also draw insights from this work. For example, the integration of reaction norms into animal welfare, evolutionary medicine and public health concepts may lead to more nuanced policies and improved treatments [77–79].

## Data Availability

The dataset and the R code generated for this study are available on Zenodo: https://doi.org/10.5281/zenodo.7018713 [80]. An earlier version of this manuscript was posted at bioRxiv: https://doi.org/10.1101/2022.04.21.489013 [81]. Additional information is available in the electronic supplementary material [82].
